# A mechanistic study identifying improved technology critical metal delamination from printed circuit boards at lower power sonications in a deep eutectic solvent

**DOI:** 10.1016/j.ultsonch.2023.106701

**Published:** 2023-11-23

**Authors:** Ben Jacobson, Shida Li, Rodolfo Marin Rivera, Paul Daly, Christopher E. Elgar, Daniel M. Mulvihill, Andrew P. Abbott, Andrew Feeney, Paul Prentice

**Affiliations:** a James Watt School of Engineering, University of Glasgow, Glasgow G12 8QQ, UK; b School of Chemistry, University of Leicester, Leicester LE1 7RH, UK

## Abstract

Deep eutectic solvents (DESs) are an emerging class of ionic liquids that offer a solution to reclaiming technology critical metals (TCMs) from electronic waste, with potential for improved life cycle analysis. The high viscosities typical of DESs, however, impose mass transport limitations such that passive TCM removal generally requires immersion over extended durations, in some cases in the order of hours. It is postulated that, through the targeted application of power ultrasound, delamination of key structures in electronic components immersed in DESs can be significantly accelerated, thereby enabling rapid recovery of TCMs. In this paper, we fully characterise cavitation in a Choline Chloride-Ethylene Glycol DES as a function of sonotrode input power, by the acoustic detection of the bubble collapse shockwave content generated during sonications at more than 20 input powers over the available range. This justifies the selection of two powers for a detailed study of ultrasonically enhanced TCM-delamination from printed circuit boards (PCBs). Dual-perspective high-speed imaging is employed, which facilitates simultaneous observation of TCM removal, and the cavitation evolution and interaction with the PCB surface. Bubble jetting is identified as a key contributor to initial pitting of the TCM layers, exposing the larger underlying copper layer, with the contributions of additional inertial cavitation-mediated phenomena such as bubble-collapse shockwaves also demonstrated as important for delamination. Optimal cavitation activity throughout the sonication then promotes etching of the copper base layer of the PCB structure targeted by the DES, liberating the overlaying TCMs in sections as large as 0.79 mm^2^. We report a thirtyfold improvement in processing time compared to passive delamination, with sonications at the lower power outperforming those at the higher power. The results demonstrate the potential for industrially scalable recovery of TCMs from the growing quantities of global e-waste, using combined power ultrasonics and DESs.

## Highlights

• Cavitation in a DES is characterised as a function of input power via detection of the acoustic emissions.
• Dual-perspective high-speed imaging provides detailed insight to cavitation-mediated TCM-delamination from PCBs.
• Sonications accelerate delamination processes by a factor of 30 over passive immersion.
• Sonications at a lower input power are more effective than those at a higher power.

## Graphical abstract

**Figure:**
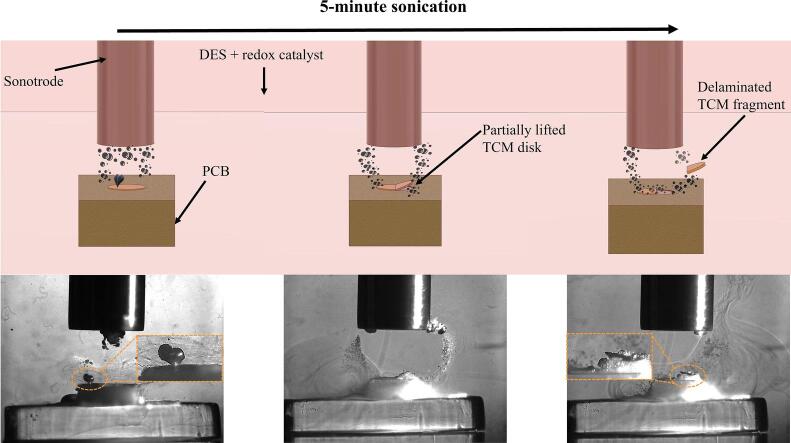

## 1 Introduction

The volume of electronic waste (e-waste) produced worldwide has reached 55 Mt, with this figure expected to rise to 75 Mt by 2030 [1]. Reports suggest that less than 20 % of global e-waste produced is properly collected and processed [1], [2]. The UK alone produced a total of 1.6 Mt of e-waste in 2019, containing *ca.* 380,000 kg of technology critical metals (TCMs) worth $148 M per annum [3].

Within this, waste printed circuit boards (PCBs) account for around 3 % of all worldwide e-waste per annum [4], [5]. Although this is a relatively small fraction of total global e-waste, PCBs contain up to 30–40 wt% of TCMs such as cadmium, chromium, copper, lead, manganese, nickel and tin [5]. Furthermore, precious metal content such as gold, silver, platinum and rare-earth elements can be found in quantities exceeding 10 times the concentration found in enriched mineral ores [6].

Traditional pyro- and hydrometallurgical processing techniques are commonly used for recovering metals from e-waste [7], [8], [9] but lack selectivity and have significant environmental impact [10]. Ionometallurgy is a promising new technique for recovering metals from e-waste [11], [12], [13], [14] using non-aqueous solvents such as deep eutectic solvents (DESs). Ionometallurgy offers distinct advantages over the traditional techniques, including much lower temperature requirements, avoidance of toxic reagents and reduced water consumption [15]. DESs are also cheap, readily available and can be adapted for selectivity of target metals [10]. Despite these advantages, current ionometallurgy approaches using DESs are limited by slow dissolution kinetics, with a recent study reporting that TCM removal from PCBs required immersion in DES for several hours [10]. The addition of redox catalysts can aid in addressing this issue and has shown utility in selectively separating metals from PCB and photovoltaic substrates [10], [16], [17], however this purely chemical-based enhancement is still limited by mass transport. DESs are characterised by high viscosity (ca 40 mPa s) [18] which is detrimental to conductivity and diffusion of metal ions.

One approach to overcoming mass transport limitations which is now demonstrating potential, is via the introduction of power ultrasonics for mechanical and chemical acceleration of ionometallurgical processes. Sonication of DESs is postulated to increase mass transport, remove passivating surface layers and promote radical formation via cavitation-mediated phenomena [19], [20], [21], [22]. Previous research has demonstrated ultrasonically-enhanced leaching of metals from ores using strong mineral acids [23] as well as in Ethaline-DES for electrochemical leaching and re-deposition of copper [24], [25]. Further studies have shown the utility of ultrasonically assisted separation of TCMs from photovoltaics and lithium-ion batteries [17], [26]. Despite the clear potential of ultrasonically enhanced ionometallurgy, little is understood of the mechanisms underpinning accelerated TCM delamination, and optimisation in terms of ultrasonic parameters has yet to be explored.

The principal objectives for the current work are two-fold: characterisation of cavitation activity in Ethaline-DES as a function of sonotrode input power, as has recently been reported for water [27], and a mechanistic investigation of TCM delamination from PCBs submerged in sonicated DES. The delivery of these objectives is critical to progress industrially scalable recovery technology. To achieve the former objective, shadowgraphic high-speed imaging (HSI) was used to characterise the evolution of distinctive cavitation structures in the high-viscosity DES, during sonication at more than 20 input powers, whilst the acoustic emissions were simultaneously sampled, with a passive cavitation detector. Results from this investigation are reported in *Supplemental Materials* and guided selection of two input powers for use in meeting the second objective of observing ultrasonically enhanced TCM delamination from PCBs. For this part of the work, a second high-speed camera was introduced to monitor the TCM surface on the PCB, during each sonication. Surface microscopy and profilometry was undertaken at regular intervals within the total sonication, to assess for TCM-layer delamination, as demonstrated in *Results*, section 3.2. Selected dual-perspective HSI data of significant delamination events at both input powers are presented in *Results*, section 3.3.

For assessing the degree of ultrasonic enhancement in the current work, the results presented below can be compared to those of Rivera *et al* [10], where passive immersion was used to reclaim TCMs from similar PCB samples. This study investigated the rate of TCM removal for the same DES as is used here, at two different concentrations of anhydrous Iron (III) Chloride oxidising agent, which chemically stimulates removal; specifically, 10 × higher and lower than the concentrations used here (see *Materials & Methods*, section 2.1). The authors reported etch rates of 24.5 and 0.96 µm/hour for these concentrations, respectively, at room temperature, with passive immersion for 8 h resulting in ∼80 % TCM removal, by area, from the PCB structure.

## 2 Materials & methods

### 2.1 Deep Eutectic Solvent (DES) preparation

The DES used throughout this study was Ethaline – a mixture of Choline Chloride (ChCl) and Ethylene Glycol (EG) in a 1:2 ratio. The DES was prepared by mixing the components at 60 °C until a colourless homogenous liquid was formed. For the delamination experiment, a redox catalyst of anhydrous iron (III) chloride (FeCl_3_) was added at a ratio of 0.1 mol dm^−3^. This concentration is 10× lower and higher than those used by Rivera et al. for passive immersion [10]. This concentration was selected to maximise chemical stimulation but reduce the opacity of the DES sufficiently, to allow HSI of cavitation development during sonication.

### 2.2 Printed Circuit Board (PCB) samples

**Fig. 1:**
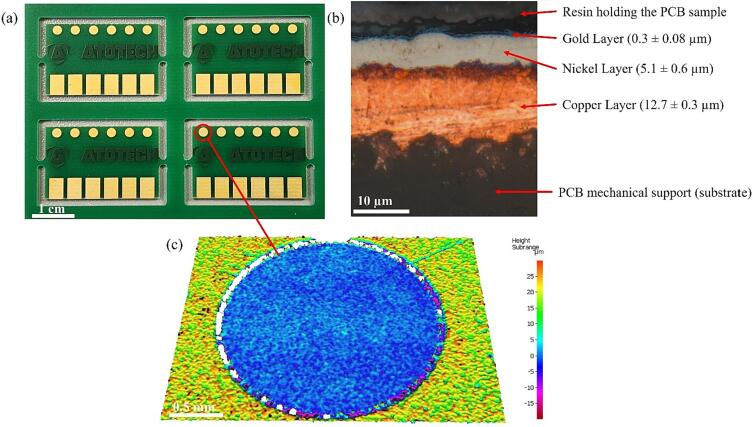
a) Gold-coated printed circuit board as received from supplier. b) reflected light image of metal layer profile for all printed features c) profilometry of (untreated) TCM disk feature, used throughout this study.

**Fig. 2:**
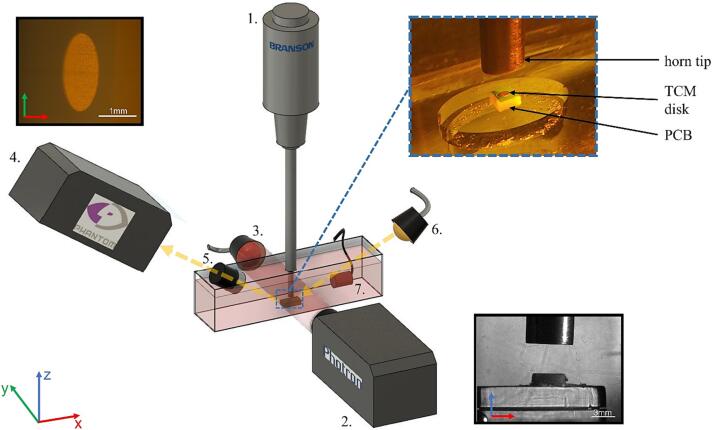
Schematic representation of the experimental setup, featuring the following components: 1. The sonotrode, the tip of which was aligned to a PCB section (inset top-right) within a custom-made tank filled with DES. 2. Photron high-speed camera used to study cavitation development in the vicinity of the tip, with pulsed laser illumination provided via a collimator lens, 3, with FOV depicted (inset bottom-right). 4. Phantom high-speed camera for direct observation of the TCM disk, with objective lens 5, and white light illumination, 6. Typical FOV as depicted (inset top-left). Acoustic detection of cavitation activity was undertaken with a swPCD, denoted by, 7.

PCB sections were diced from a larger board, as received from the supplier (Atotech UK Ltd), Fig. 1, into uniform sections, each containing a single 2 mm Ø TCM disk. Each TCM disk has the same size and metal profile, Fig. 1. The PCB was then fixed onto a Perspex disk within the centre of tank for repeatable positioning (Fig. 2 inset top right, and also visible in the Photron field-of-view, bottom right).

### 2.3 Characterisation of cavitation activity in DES

For the results characterising cavitation in DES as a function of input power, *Supplemental Materials* and *Results* section 3.1, shadowgraphic HSI and acoustic emission data was collected from five 200 ms sonications, sampling input power in 5 % increments, or less.

#### 2.3.1 High-speed imaging

HSI of the cavitation activity in the vicinity of the sonotrode tip was undertaken with a Fastcam SA-Z 2100 K (Photron, UK) (*component 2*, Fig. 2). Illumination was provided via synchronous 10 ns laser pulses at 640 nm (CAVILUX Smart, Cavitar, Finland), coupled to a liquid light guide and a collimating lens (*component 3*, Fig. 2). In addition to setting the effective temporal resolution (the duration of frame capture), this illumination configuration facilitates shadowgraphic HSI such that bubble-collapse shockwaves may be directly imaged, via refractive index variations imposed by the pressure transient of the propagating shockwave [27], [28], [29], [30]. Imaging was undertaken through a macro-lens (Milvus 100 mm f/2M, Zeiss, Oberkocken, Germany), over 486 × 324 pixels, providing a spatial resolution of 39 μmpixel^−1^, with the field-of-view (FOV) as shown in Fig. 2 (inset, bottom-right). For the characterisation of cavitation in the DES, imaging was obtained at 80 kfps over a duration of approximately 5 s. This HSI was sufficient to track tip-vibration throughout sonications and no significant variations in tip-vibration amplitudes were observed during sonications.

#### 2.3.2 Passive cavitation detection

Acoustic detection of cavitation emissions was undertaken with a bespoke, in-house fabricated passive cavitation detector, based on 110 μm thick PVDF and designed for high-sensitivity to bubble-collapse shockwaves [31].The shockwave passive cavitation detector (swPCD) used in this study has an active element 10 mm in diameter, and was mounted on an x, y, z manipulator for positioning within the tank, Fig. 2, to detect emissions orthogonally (in the *x-direction*), with respect to the sonotrode probe (*z-direction*).

The swPCD was connected to an oscilloscope (Tektronix 5 series, Berkshire UK) for data collection at 25 × 10^6^ samples/s. Acoustic emissions were recorded for a total duration of 200 ms, triggered ∼4 s into the sonication. Emission data was collected in millivolts (mV), as detected by the hydrophone. A filtering protocol was applied to reduce noise (low-pass < 10 MHz) and *f_0_* (high-pass > 20 kHz), revealing shockwave content for presentation in the voltage–time domain, *Supplemental Materials*. Data is also presented in the frequency domain, via application of a fast Fourier transform and Blackman window over the duration of the time signal (MATLAB, MathWorks).

Time-averaged shockwave content is quantified by the root mean square of the voltage (V_RMS_), over the five 200 ms duration samples per power, for > 20 input powers, as described in the *Supplemental Materials* and summarised in *Results*, section 3.1.

### 2.4 The experimental setup

The characterisation of cavitation activity experiments were carried out with the same equipment as the PCB delamination experiments, with two exceptions: (i) the lack of PCB in the former experiment and (ii) the addition of a second high-speed camera for the latter experiment, described below.

All results presented were obtained with a 450 W sonotrode (Digital Sonifier, Branson 450) operating at 20 kHz through a 230 mm long tapered Ti probe with a 6.4 mm diameter tip (*component 1*, Fig. 2). Input power for the sonotrode is entered as a percentage value on the control console, with a minimum of 10 % and programmable in 1 % increments. The sonotrode tip was positioned ∼4 mm above the PCB, at a consistent immersion depth of ∼40 mm.

### 2.5 Technology critical metal (TCM)-disk delamination

For the TCM delamination investigation, dual-perspective HSI was conducted with the Photron camera as described previously in *Results* section 2.3.1 but at a reduced frame rate of 8 kfps (giving a resolution of 1024 × 758 pixels), to facilitate longer record duration. A Phantom high-speed camera recording from a second imaging perspective described in section 2.5.1, was also incorporated.

#### 2.5.1 Dual-perspective high-speed imaging

A Phantom V710 (Vision Research, New Jersey USA, *component 4*, Fig. 2) high-speed camera was used to directly observe the TCM-disk surface during sonication. Imaging from this perspective was undertaken at 1 kfps, through a long working distance 5× microscope objective lens (0.14NA Mitutoyo, Japan) (*component 5*, Fig. 2), dipped into the DES at an angle of ∼40° above the horizontal. This was necessary to observe the disk beneath the sonotrode tip. Illumination was provided by a 150 W halogen lamp, coupled to a liquid light guide and a collimating lens (Thorlabs, UK) (*component 6*, Fig. 2). The FOV from this angled imaging perspective is shown in Fig. 2 (inset top-left), where the gold layer of the TCM-disk, Fig. 1 (b) provides the reflective surface, with a line focus across the *y*-direction such that the proximal and distal edges of the disk are slightly out of focus.

### 2.6 Data collection

PCB samples were removed after each minute of accumulated 10 s sonication, washed in deionised water and scanned with an Alicona Infinite Focus G4 3D surface profilometer (Bruker-Alicona, Graz Austria). Surface microscopy and profilometry was obtained through a 5× lens (Bruker-Alicona) for every sample, with additional higher magnification (20×) profilometry obtained for regions of notable delamination. 2D surface microscopy across each sample at 1-minute intervals allowed quantification of delamination evolution over time via a bespoke imaging thresholding macro in ImageJ (National Institute of Health, Wisconsin USA).

Sonications were initiated manually from the control console of the sonotrode, at defined input powers. The remaining instrumentation was synchronised via electronic triggering controlled from a signal generator (DG4102, Rigol Technologies, Beijing China). For TCM-delamination, Photron and Phantom HSI were synchronised to record the entire 10 s sonications with both cameras triggered immediately before the initiation of sonication. A total of five PCB samples were analysed at each input power, for a total accumulated sonication time of 5 minutes.

## 3 Results

**Fig. 3:**
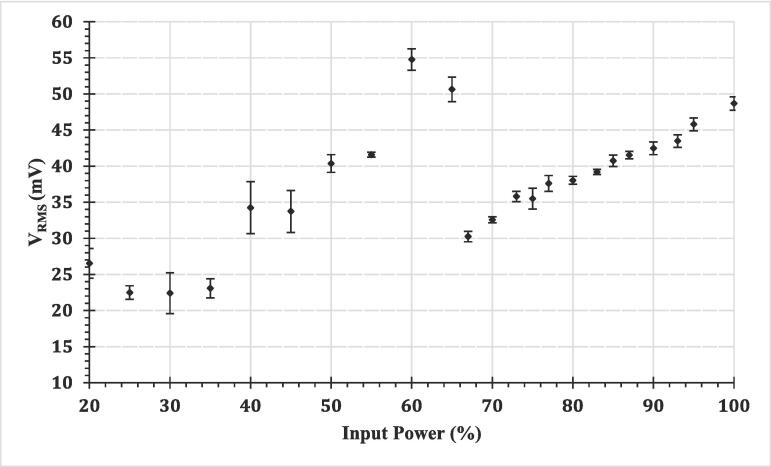
Mean V_RMS_ from five 200 ms sonications at each input power, of the signal detected by the swPCD during sonications in DES.

The results below are organised as follows: section 3.1 presents the characterisation of the cavitation behaviour in DES, with V_RMS_ of the swPCD detections plotted as a function of input power plot across the range available with the sonotrode. The data underpinning this plot are described and represented in *Supplemental Materials.* Fig. 3 guided selection of two input powers for taking forward to the overview of TCM-delamination performance, section 3.2, and HSI of delamination events, section 3.3.

section 3.2 presents the 2D microscopy and 3D profilometry observations of the delamination of five PCB samples, at 1-minute intervals throughout the 5-minute total accumulated sonication time, as an overview of the delamination performance at each of the selected input powers.

In section 3.3, dual-perspective HSI of several key TCM-delamination events at each selected input power is presented. Comparison between the cavitation observed at two input powers is made, facilitating mechanistic interpretation of the delamination performance.

### 3.1 Characterisation of cavitation activity in DES

Fig. 3 is a plot of V_RMS_ versus input power for cavitation generated in Ethaline-DES, with each data point representing an average of five sonications, at each input power sampled. Representative data and a full explanation of the plot is available in *Supplemental Materials*. Briefly, however, V_RMS_ of the filtered cavitation emission signal is taken as representative of the average shockwave content within the signal generated during a sonication. We have previously shown that for sonications in water (which has also been reproduced with the current experimental configuration in *Supplemental Materials*, for comparative purposes), V_RMS_ has a non-linear dependence on input power. Moreover, it was suggested that the structure of a V_RMS_ versus input power plot can be used as a simple guide to selecting the optimal input power, for any sonochemical application mediated by cavitation bubble collapses [27].

The results shown in Fig. 3 indicate that a sonication in DES at 60 % input power generates almost double the shockwave content that is generated during a sonication at 70 % input power. As described in *Supplemental Materials*, this is because a 70 % input power sonication generates cavitation that is transitioning between orders of subharmonic response (specifically, *f_0_*/*m*, where *m* increases through positive integer values, with increasing power). The cavitation at such a transitioning power (or tip-vibration amplitude), does not oscillate and collapse as smoothly or periodically as that at the lower power, generating irregular and intermittent shockwave emissions, which is reflected in the lower V_RMS_ at the higher power (see *Supplemental Materials*). For this experimental configuration, optimal cavitation activity was observed at 60 % input power, with periodic shockwaves at an *f_0_/2* subharmonic response. For different experimental arrangements, full acoustic characterisation would be required to characterise the cavitation generated for that system.

The results described in *Supplemental Materials* also suggest that distinctive cavitation structures develop at different powers. Notably, at 60 % input power, a bulbous bubble structure extends through the DES, as has been previously reported for sonotrode sonication in high viscosity liquids [32] and is sustained for most of a 10 s sonication. In contrast, at the higher powers of 70 % and 90 %, although a bulbous structure develops earlier in the sonication, it also recedes back into a conical structure in contact with the tip (reminiscent of sonotrode cavitation in lower viscosity liquids, such as water), more rapidly at the higher powers.

In the TCM delamination results below, we demonstrate, for the first time, the effectiveness of using this characterisation approach to selecting optimal input powers. Specifically, observations were conducted at 60 % and 90 % input powers, such that the difference between the input powers may be taken as significant, and the delamination performance at each power is assessed. The selected input powers at 60 % and 90 %, as justified by this characterisation approach, are specific to the experimental configuration used in this study and are not likely to be directly transferrable to other ultrasonic systems.

### 3.2 Overview of TCM-delamination performance at two selected input powers

In this section, surface microscopy and profilometry of the TCM-disk, taken at 1-minute intervals throughout the total accumulated sonication time of 5 min, at each of the input powers identified in the previous section, are reported. Specifically, each PCB sample was exposed to six 10 s sonications (to facilitate dual-perspective HSI of the cavitation activity during the entire sonication and any delamination events that occurred, observations from which are reported in *Results* section 3.3, below), removed for microscopical and profilometry examination, then returned to the DES tank for a further six 10 s sonications. The dataset consists of five PCB samples each at 60 % and 90 % input power, with a further five samples sonicated continuously for 5 min in DES, as a baseline delamination assessment for the effective 10 s pulsing required to capture the HSI at each power. In addition to the dual-perspective HSI of delamination mechanisms (*Results* section 3.3), we seek to assess the utility of characterising the cavitation in DES under the current experimental configuration for identifying effective powers at which to administer sonications.

**Fig. 4:**
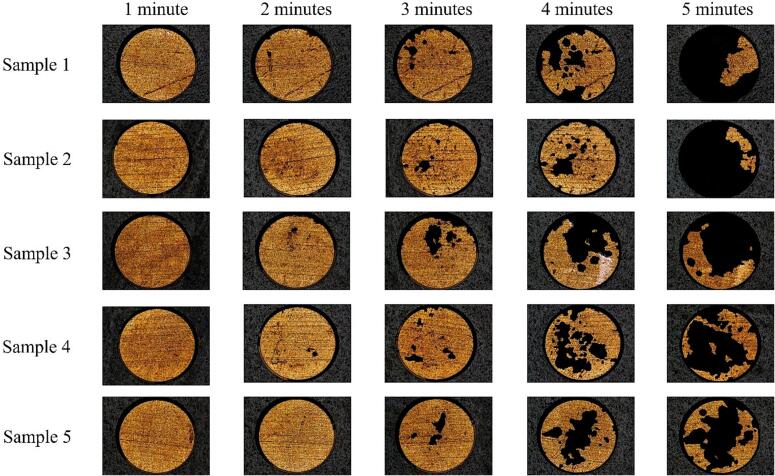
Surface microscopy of TCM-disks from five PCB samples, taken at one-minute intervals (after 6 × 10 s sonications), over a total sonication of five minutes (30 × 10 s sonications) at 60 % input power, demonstrating the accumulated delamination. Scale is provided by the 2 mm Ø disk.

**Fig. 5:**
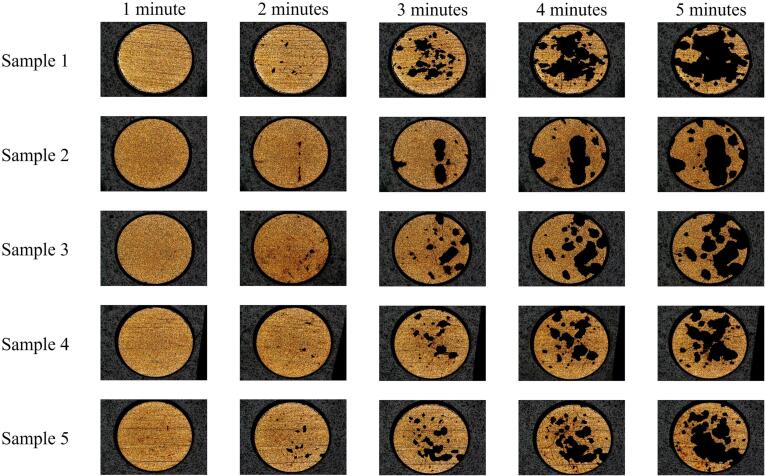
Surface microscopy of TCM-disks from five PCB samples, taken at one-minute intervals (after 6 × 10 s sonications), over a total sonication of five minutes (30 × 10 s sonications) at 90 % input power, demonstrating the accumulated delamination. Scale is provided by the 2 mm Ø disk.

Fig. 4, Fig. 5 are Alicona surface microscopy images of the TCM-disk at 60 % and 90 % input powers, respectively. The metal layer profile of Fig. 1 (b) indicates it is the gold layer that is visible, as the top reflective surface.

**Fig. 6:**
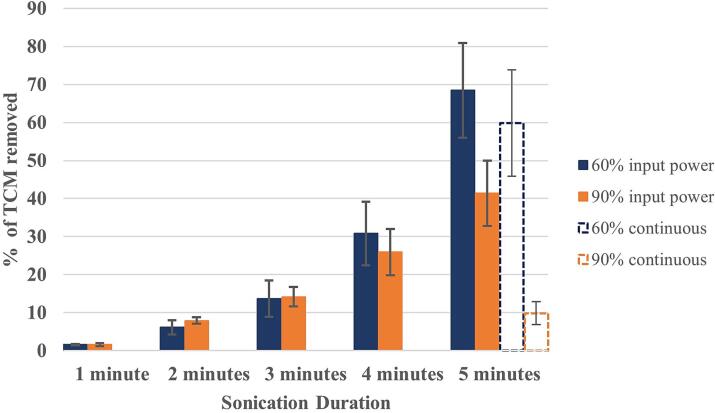
Histogram of percentage TCM removal vs sonication time presented as an average of the five samples (± standard deviation) at 60 % input power (blue) and 90 % input power (orange). Average delamination for samples continuously sonicated for five minutes are also presented (dashed).

Both Fig. 4, Fig. 5 indicate negligible (observable) delamination over the first minute of sonication. Even sample 1, which features a pre-existing score feature, which may be expected to provide a discontinuity on the surface and act to nucleate and promote cavitation activity, does not show any sign of early delamination during the first minute of sonication. We note low levels of apparent TCM delamination after the first minute of sonication in Fig. 6, which quantifies the average percentage of TCM removal for all samples at each power, at each 1-minute sonication intervals. The ∼2 % TCM removal is at least in part attributable to thresholding applied during implementation of the ImageJ macro. By the end of the second minute of 10 s sonications, however, most samples at both input powers exhibit some form of initial delamination feature, typically an almost circular hole or pit, approximately 40–50 μm in diameter. Fig. 6 confirms that there are a few more of these features at the higher input power of 90 %. After the third minute of sonication, samples are either exhibiting many more of these features, or the features formed during previous sonications have been significantly widened. During the last two minutes of sonication, individual holes continue to widen and merge. The main finding, however, is that after the fifth minute, the percentage of TCM removal is 68 ± 13 at 60 % input power, comparing to 40 ± 10 at 90 % power.

Fig. 6 also includes percentage TCM removal for a continuous 5-minute sonication at the two input powers (without accompanying HSI or, evidently, minute-by-minute surface microscopy). TCM delamination is reduced for continuous sonication at both powers, notably so for 90 % input power, with only 10 ± 3 % removal. Continuous sonications were not directly observed due to limitations of the HSI record duration. Therefore, direct comparison of TCM delamination to that observed by the shorter 10 s sonications cannot be made.

**Fig. 7:**
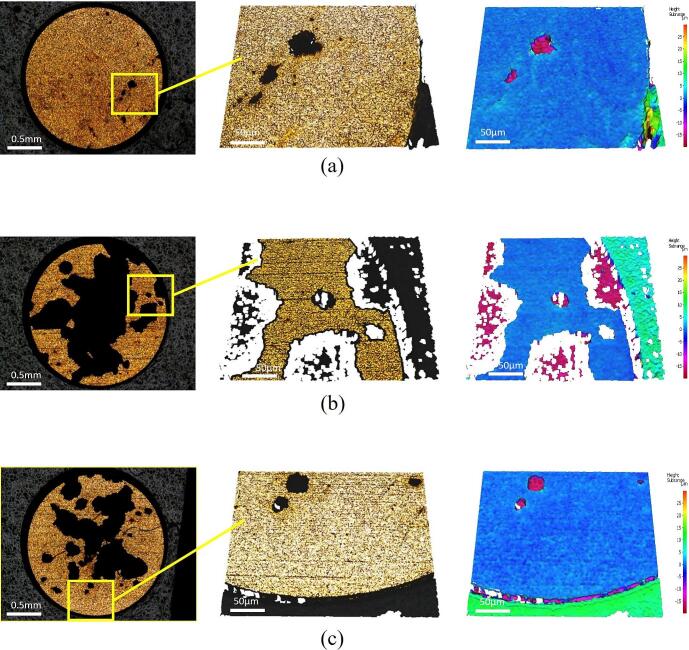
Profilometry of removal features on the TCM-disk at 20× magnification for (a) sample 3, after the second minute of sonication at 90 % power, (b) sample 5 after the fifth minute of sonication at 60 % power and (c) sample 4 after the fifth minute of sonication at 90 % power.

To further investigate and characterise the removal features, profilometry was conducted on every sample while it was mounted on the microscope, between 1-minute sonications. Fig. 7 displays profilometry data at various sonication intervals, and at both input powers.

All removal features investigated during this study had a depth of ∼18 μm, indicating complete removal of the TCM layers. We did not observe any evidence of layer-by-layer removal, including around the edges of the features.

### 3.3 Dual-perspective HSI of TCM-delamination

This *Results* section presents combination Phantom and Photron imaging of specific delamination events on single samples, captured by both cameras, at 60 and 90 % input power. It should be noted that these observations have been selected from an extensive HSI dataset of many delamination events, recorded throughout the course of this study. The observations presented were chosen as being representative of delamination events for each power, and stage, of the sonication, as stated – and with TCM removal observable from both HSI perspectives. Moreover, the evolution of cavitation structures, observed from the Photron HSI perspective, are typical of that which occurred at each power, and stage, of the sonications.

**Fig. 8:**
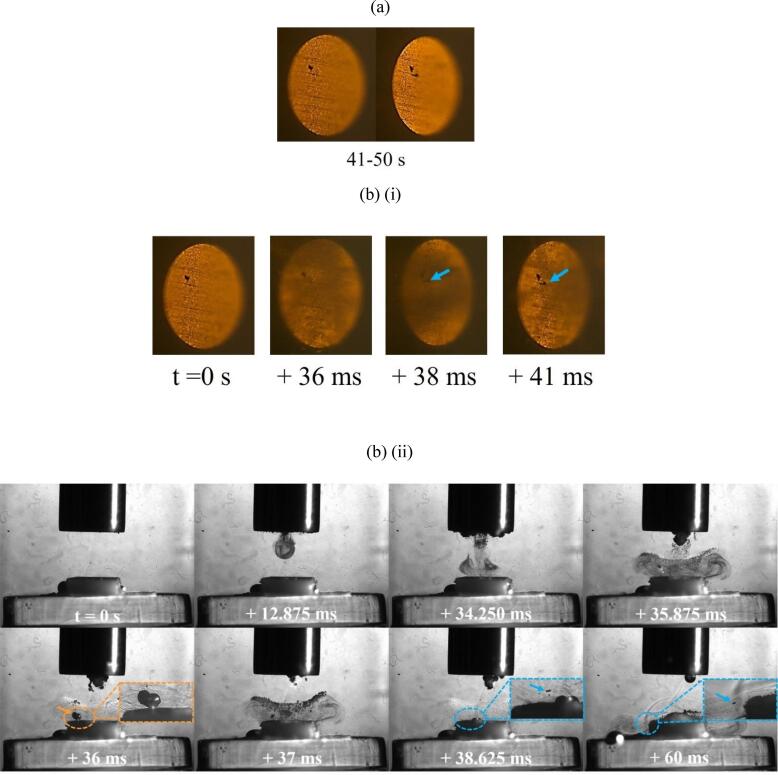
(a) Single before and after images of an early stage TCM delamination event from the Phantom camera perspective, occurring during the 41–50 s sonication of the second minute at 60 % input power, for sample 4. (b) Combination (i) Phantom and (ii) Photron frames from an HSI sequence of specific delamination events during the 41–50 s sonication. A jetting bubble at 36 ms (arrowed orange and zoomed inset) dislodges a TCM fragment (arrowed blue within the zoomed insets at 38.625 and 60 ms). Image sequences are available in movie format as supplementary material. Scale provided by (i) the 2 mm Ø TCM disk, (ii) the 6.4 mm Ø tip.

HSI results figures are presented as follows, taking Fig. 8 as an example: (a) presents single frame captures of the TCM-disk taken with the Phantom camera *before* and *after* a 10 s sonication, in this case the 41–50 s sonication of sample 4, during the second minute, at 60 % input power. The overall delamination morphology from the surface microscopy data of Fig. 4, Fig. 5 can be cross-referenced to the Phantom *before* and *after* captures, for any given sample at each stage of sonication at either power, throughout the HSI figures presented below.

Fig. 8 (b) (i) are frames extracted from the Phantom high-speed sequence captured during the sonication, here showing the appearance of the hole features typical of the earliest observed delamination events, for all PCB samples. For all frames extracted from a HSI sequence (both Phantom and Photron), *t = 0 s* refers to the start of that specific 10 s sonication, with all other timings given relative to that. The cavitation activity between the sonotrode tip and PCB surface can somewhat obscure the disk imaging from this perspective (at 36 and 38 ms of Fig. 8 (b) (i)), although the development of delamination is often still apparent.

Fig. 8 (b) (ii) are frames extracted from the Photron imaging sequence, corresponding to the sampled Phantom imaging of Fig. 8 (b) (i), showing the specific cavitation activity around key moments of delamination. The right-hand side of many objects in the Photron imaging, including bubbles and the sonotrode tip, are brighter due to the continuous white light illumination for the Phantom imaging perspective, Fig. 2, which is reflected into the Photron imaging perspective.

Fig. 8 (a) demonstrates that a small individual hole was inflicted onto the TCM-disk, during the 41–50 s sonication, ∼100 µm from a similar looking feature from a previous sonication. This HSI sequence indicates this second hole occurs ∼36 ms into this 10 s sonication and is observable at 41 ms, through the cavitation activity. The Photron imaging of Fig. 8 (b) (ii) captures a bubble exhibiting an involution (arrowed orange at 36 ms), indicative of jet-formation which would be expected in such close vicinity to a rigid surface, under acoustic driving [20], [33]. Subsequent images from the sequence capture a small fragment of material (which can be distinguished from the bubbles because it does not oscillate under the ultrasonic driving, arrowed blue within the zoomed insets, throughout) originating from the region of the TCM-disk that was impacted by the bubble-jet, around 36 ms. The additional contribution from surface interacting inertial bubbles and bubble-collapse shockwaves previous to the observed jet, are also likely to contribute to the delamination of the TCM-disk. Acoustic streaming under the tip of the sonotrode, which is often revealed by vortex-like structures in the bubble mist (as also observed in *Supplemental Material*) acts to propel the TCM fragment away from the centre of the disk and over the edge of the PCB section, at 60 ms.

**Fig. 9:**
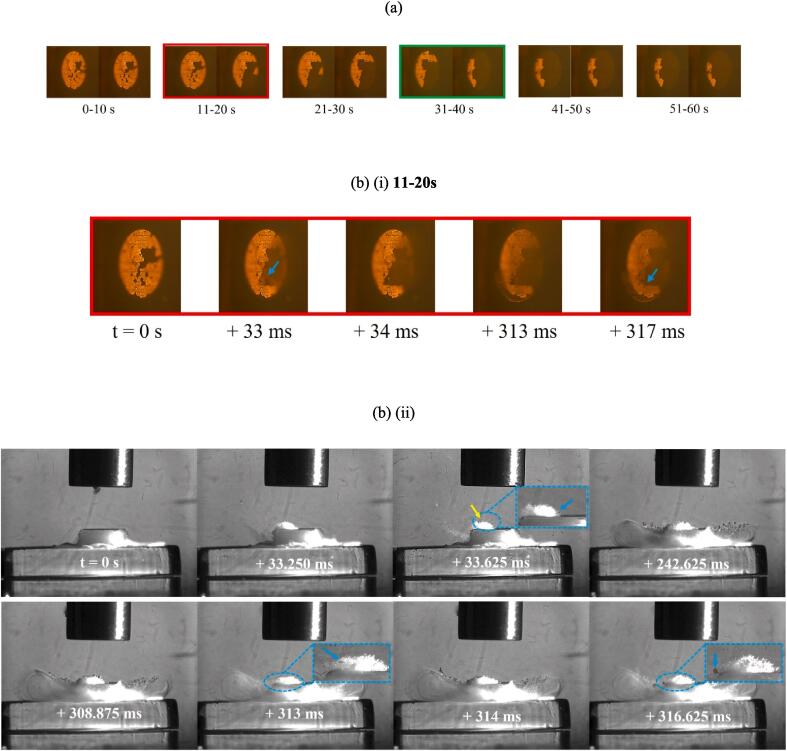

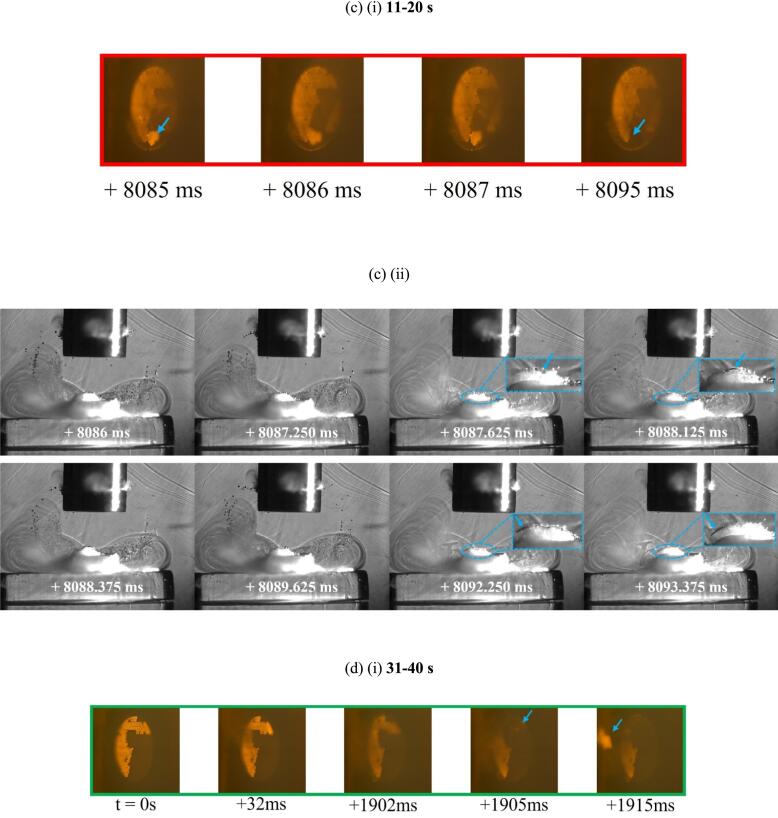

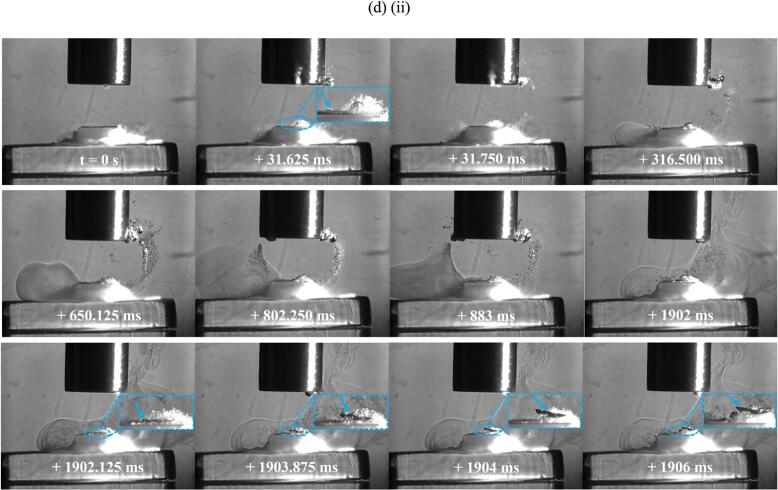
(a) Single images of the TCM disk from the Phantom camera perspective, before and after each of the 10 s sonications, from the fifth minute at 60 % input power for sample 2. (b)–(d) Combination (i) Phantom and (ii) Photron frames from HSI sequences, of specific delamination events at different points during the sonication. Bubble collapse shockwaves are arrowed yellow, TCM fragments within zoomed insets arrowed blue, throughout the Photron imaging. Image sequences available in movie format as supplementary material. Scale provided by (i) the 2 mm Ø TCM disk, (ii) the 6.4 mm Ø tip.

Fig. 9 is representative dual perspective HSI taken during the fifth minute of sonication of sample 2, at 60 % input power. It contains a significant amount of data – and is representative of much more – necessary to adequately represent the main events occurring within the complex cavitating environment, over an extended time. In the following text, only the salient features are highlighted.

As is clear from Fig. 4, the fifth minute of sonications at 60 % input power caused widespread delamination for all samples. Fig. 9 (a) demonstrates TCM delamination from sample 2 totalling an area ≈ 0.79 mm^2^ (∼25 % of the total TCM disk area) occurred during the 11–20 s sonication, of the fifth minute, with less than 20 % of the TCM disk remaining after the 31–40 s sonication.

**Fig. 10:**
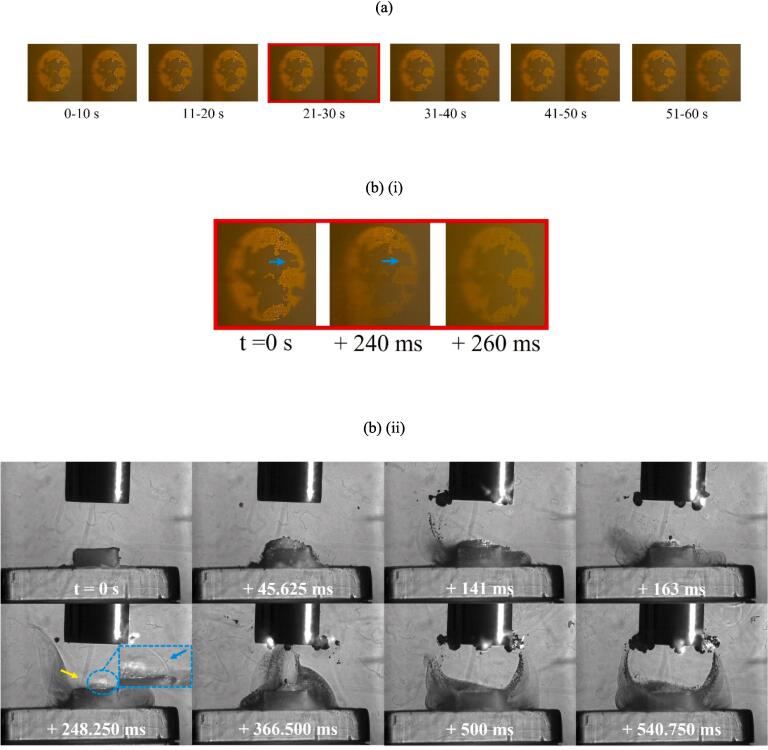

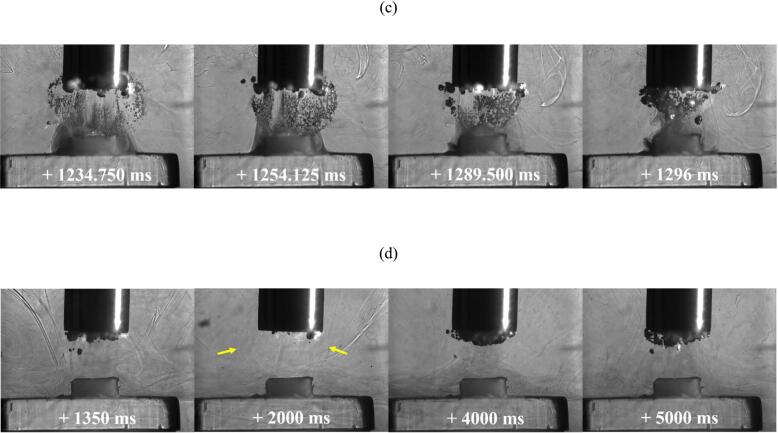
(a) Single frame captures of the TCM disk from the Phantom camera perspective, before and after each of the 10 s sonications, from the fifth minute at 90 % input power, for sample 1. (b) (i) Phantom frames from a HSI sequence, of specific delamination events during the 21–30 s sonication. (b) (ii) corresponding Photron frames for the HSI sequence of specific delamination event detected by the Phantom. (c) Change in the cavitation structure after ∼1.2 s, with the bubble activity receding from the PCB over 60 ms. (d) complete transformation of the cavitation activity after the first ∼1.3 s, beyond which a primary cluster close to the sonotrode tip dominates for the remainder of sonication duration. Bubble collapse shockwaves are arrowed yellow, TCM fragments within zoomed inset arrowed blue, throughout the Photron imaging. Photron imaging sequence available in movie format as supplementary material. Scale provided by (i) the 2 mm Ø TCM disk, (ii) the 6.4 mm Ø tip.

Fig. 9 (b) (i), (c) (i) and (d) (i) are frames extracted from the Phantom high-speed sequence capturing three distinct delamination events that occurred during this minute of accumulated sonications. It is the corresponding Photron imaging of Fig. 9 (b) (ii), (c) (ii) and (d) (ii), however, that capture the key features for the cavitation at 60 % input powers, distinguishing it from that at 90 % (presented below, Fig. 10). Specifically, a sustained, tightly packed and vigorously cavitating cluster of cavitation bubbles is shown to remain in contact with the PCB surface throughout all 10 s sonications at 60 % (this also occurred later in the sonication represented by Fig. 8). Another interesting feature is the bubble filaments of the bulbous structure, that curve downwards from the edges and sides of the sonotrode tip, toward the PCB sample. This bulbous structure has been reported previously for sonications in highly viscous fluids [32], [34], [35] and also features in the cavitation characterisation in DES results, of *Supplemental Materials.* For the TCM delamination observations of Fig. 9, the curving bubble filaments appear to feed the cavitation cluster that persists on the PCB surface, throughout sonications at 60 % input power.

Fig. 9 (b) (i) demonstrates delamination of a small single TCM fragment, occurring during the initial 317 ms of the 11–20 s sonication (red box, Fig. 9 (a)). At 33 ms, a fragment of TCM is prised upwards from the PCB, although not fully delaminated. Fig. 9 (b) (ii) of the corresponding Photron HSI sequence show the TCM fragment emerging from the persistent cavitation cluster at the PCB surface at 313 ms. Bubble collapse shockwaves (arrowed yellow throughout), such as at 33.625 ms, indicate that the surface cluster is cavitating vigorously. Sequences from both cameras are available in movie format as supplementary material *files..*

Fig. 9 (c) are dual perspective HSI from later in the same 10 s sonication, with a further TCM delamination event occurring shortly after 8 s into the sonication (i). Fig. 9 (c) (ii) demonstrates that the cluster at the PCB surface has been sustained throughout the sonication. The imaging from both perspectives again strongly suggest a gradual prising of a much larger TCM fragment than that in Fig. 9 (b) earlier in the sonication, from the surface of the PCB over several ms, before full delamination to removal is apparent, in the Phantom imaging between 8087 and 8089 ms, and in the Photron imaging at 8092.250 ms.

Fig. 9 (d) highlights another significant TCM removal event from sample 2 at 60 % input power during the 31–40 s sonication of the fifth minute. Additional Photron imaging is included in Fig. 9 (d) (ii) to demonstrate that the prising and delamination of a large TCM fragment can take some time to fully separate from the PCB substrate. The Phantom imaging of Fig. 9 (d) (i) suggests the prising of this fragment (∼20 % the total TCM-disk area) starts at 32 ms, with the Photron imaging of Fig. 9 (d) (ii) confirming the surface cluster formed on the PCB around this time. For almost 2 s, the fragment (still attached to the substrate) is ‘jostled’ by the cavitation (best appreciated in the movie version of the Phantom HSI, available as supplementary material. The fragment is finally liberated at ∼1902 ms, with the removal process well captured by the Photron imaging of Fig. 9 (d) (ii), from 1902 to 1906 ms.

Fig. 10 (a) presents single frame captures from the Phantom camera of delamination of the TCM-disk at 90 % input power over the fifth minute of sonication, on sample 1. Fig. 10 (b) presents early (i) Phantom and (ii) Photron image sequences extracted from the 21–30 s sonication, corresponding to TCM delamination. Fig. 10 (c) and (d) present frames extracted from the Photron HSI sequence depicting the evolution of the cavitation development beyond the first second of sonication and, in particular, highlight the differences for the cavitation that develops during sonications at 60 % input power, Fig. 9.

Fig. 10 (a) demonstrates that the full extent of delamination during the fifth minute of sonication is lower than that at 60 % power, with delamination primarily involving very small fragments of TCM and enlargement of previous pits, observed in the 21–30 s sonication, Fig. 10 (b) (i). No large fragments, comparable to those observed at 60 % power, were observed at 90 % power. Fig. 10 (b) (ii) represents the cavitation development for the first 540 ms. Over this duration, the cavitation is similar to that observed throughout the 60 % power sonications, with bubble filaments streaming towards the PCB surface and a large bubble cluster directly on the TCM-disk. Larger bubble clusters can also be observed attached to the horn tip. At 248.250 ms a small fragment of TCM is shown being removed from the PCB surface, Fig. 10 (b) (ii), zoomed inset, visible as the bubble cloud is undergoing a collapse phase, evident by the bubble collapse shockwave and corresponding to the observed delamination from the Phantom perspective (b) (i). Fig. 10 (c) demonstrates the recession of the bubble activity back towards the sonotrode tip, with individual bubble clusters appearing larger in size, and more densely compacted, than earlier in the sonication, and at a comparable time in a sonication at 60 % power, Fig. 9 (d) (ii). Beyond ∼1350 ms the bulbous cavitation structure and direct bubble/PCB contact has been entirely removed, and the sonication is reminiscent of a single tightly packed bubble cloud oscillating at the horn tip, with smaller satellite clouds detached from the main bubble cluster, Fig. 10 (d). This behaviour is equivalent to that observed in the acoustic characterisation in DES (*Supplemental Material, Fig. S.7)*. We note that no TCM delamination is observed beyond the first ∼1 s of sonication at 90 % power during any of the 1-minute intervals, suggesting that the lack of direct bubble/surface contact inhibits delamination. Image sequences for Fig. 10 are available in movie format as supplementary material*.*

## 4 Discussion

As reviewed in the *Introduction*, recycling of e-waste is an existing and rapidly growing problem for today’s consumerist society. In addition to TCM delamination from PCBs, this includes reclamation of precious and technology critical metals from aged photovoltaics and thermoelectric generators [13], [17]. Here, we present the first study investigating the mechanisms that underpin ultrasonically enhanced TCM delamination from PCBs, mediated by cavitation activity in a DES that is chemically tailored to target copper.

The main finding from the ultrasonics perspective is that, in the present conditions, sonications at a lower power are more effective than those at a higher power. The dual perspective HSI observations indicate that this occurs because the lower, 60 % input power, develops a bulbous cavitation structure which sustains an active cavitation cluster, maintaining contact with the surface of the PCB throughout the duration of a sonication. At the higher 90 % input power, this cavitation structure recedes back to the sonotrode tip earlier in the sonication – likely due to changes in liquid properties, including viscosity – removing the cavitation from the PCB surface and protecting it from any significant enhancement of delamination, for the majority of the sonication. This observation of improved effectiveness at 60 % input power is specific to this experimental arrangement. The identified optimal power will be dependent on a number of parameters such as tip diameter, liquid height and immersion depth, which have recently been demonstrated to influence the generation and size of cavitation zones in water [36], [37] and viscous liquids [38]. The values of input power identified for delamination assessment will not translate to other ultrasonic systems. Rather the cavitation activity should be assessed via the acoustic emissions across a range of input powers, as described in the supplemental materials for this experimental configuration, for guiding selection of optimal powers in other systems.

The bulbous bubble structure that determines these effects has been reported previously for sonotrode sonications in viscous liquids. *Thiemann et al* and *Eddingsaas & Suslick* [32], [34] reported very similar cavitation structures, via sonoluminescence observations in sulphuric acid, with the former also highlighting bubble jetting for the cavitation behaviour at the extremities of the bubble cloud. Our investigations suggest that a combination of consecutive bubble-collapse shockwaves and this jetting activity is important for initial pitting of the TCM disk, creating nucleation sites through which the surface cluster generated and sustained, optimally during sonications at 60 %, can penetrate. The latter study describes a bulbous globe of luminescence below the sonotrode tip, which receded to a cone shaped structure in contact with the tip, with increased acoustic intensity [32], [34], in agreement with our observations at the higher input power of 90 %, producing less effective cavitation for TCM-delamination. *Tzanakis et al* also reported a similar structure via HSI of cavitation in glycerine along with a circulating pattern of symmetrical vortices, such as that observed in this study, via the bubble mist generated in the DES [35]. Generally, the formation of the bulbous structure in liquids of higher viscosity, rather than the better-known conical structure directly beneath the tip in less viscous liquids (including water), appears to be poorly understood, and is worthy of future investigation including via simulation and modelling.

The selection of the two input powers for delamination observations was guided by a detailed characterisation of cavitation in DES, across the range of powers available, Fig. 3 and *Supplemental Materials*, based on acoustic detection of the cavitation emission signal. Specifically, the structure of the V_RMS_ versus input power plot indicates a much higher bubble collapse shockwave content (with most sonochemical applications recognised as mediated by bubble collapses), for cavitation in DES at an input power of 60 %, compared to any higher input power. The results of the *Supplemental Materials* suggest that this is due to cavitation oscillating cleanly and regularly in response to the tip-oscillation [27] and could also be related to the sustained bulbous structure formed in the DES at this power. The optimum may be different in other conditions, whereby full characterisation by acoustic detection would be required to identify potential optimal driving parameters.

*Rivera et al.* have previously demonstrated the utility of using DESs to separate TCMs from PCB substrate in silent/passive immersion conditions, noting major limitations in etch rate associated with mass transport limitations due to the viscosity of the liquid [10]. Here, through the addition of power ultrasound-generated cavitation, we have demonstrated removal of TCMs from the PCB substrate over 30 × faster.

Guided by the characterisation of cavitation in DES, *Supplemental Materials* and *Results* section 3.1, we further demonstrate that sonication of the PCB samples at a lower power of 60 % resulted in a >27 % removal of TCM compared to higher power (90 %) sonication.

We also note, however, that ultrasonically enhanced TCM delamination is not a continuous process. Rather, metal layers are removed in sections, observed in this study as initial pitting removing small fragments, with larger fragments delaminated later into sonication. As such, continuous etch rates are not suited for reporting ultrasonically enhanced delamination. Notably, the most significant delamination from the samples observed here occurred during the fifth minute of the sonications at 60 % input powers.

The DES targets and solubilises the copper layer, via oxidation in the presence of FeCl_3_, and the HSI indicates the most effective cavitation for TCM delamination occurs directly on the PCB surface. The redox chemistry of the iron catalyst can occur on the whole of the gold surface and electrons are removed from the copper layer leading to localised copper corrosion. The cavitating bubble clusters penetrate the space created by the rapid copper solubilisation, beneath the gold/nickel layers, undercutting and prising fragments of TCM from the PCB substrate. The surface microscopy and profilometry further suggest that all TCM layers are removed from the PCB substrate, with little remaining residual copper layer observed between sonications. Further processing of delaminated material would therefore be required for subsequent separation of TCM layers, once delaminated from the PCB.

Generally, for sonochemical applications, parameter optimisation is a significant challenge, with a large parameter-space available for exploration (non-exhaustively: transducer source, configuration, frequency and power of operation, and pulsing protocols). Here, we have demonstrated that cavitation characterisation for optimisation within any liquid in terms of power consumption, nominally with any source, may be easily assessed, via determining the level of shockwave content within the emission signal. We speculate that this should also apply to identifying the optimal protocols for pulsed sonications, specifically; bubble collapse shock wave content as measured by V_RMS_, could be used to determine the most efficient temporal administration of acoustic power, as well as the most effective power of sonication for a given liquid, generally.

Future work will involve assessing the effect of varying temperature of the DES during sonication. Altering temperature will have some effect on cavitation activity as well as relevant liquid properties such as viscosity [10], which has recently been demonstrated in ionic liquids [39]. Development of the cavitation structures during initiation of sonication with regard to temperature variations will be studied*.* Furthermore, the introduction of flow through forced convection and renewal of DES during sonications will be investigated. We observed a reduced effectiveness of the process at both powers, when samples are continuously sonicated for 5 min, as opposed to in 10 s intervals, used here to accommodate the dual-perspective HSI. A full exploration of pulsing, including optimal duty cycle, pulse duration and pulse interval could significantly improve the delamination efficiency, both in terms of power consumption and speed of process.

## CRediT authorship contribution statement

**Ben Jacobson:** Conceptualization, Methodology, Data curation, Validation, Formal analysis, Investigation, Writing – original draft, Writing – review & editing. **Shida Li:** Data curation, Investigation. **Rodolfo Marin Rivera:** Investigation, Resources, Writing – review & editing. **Paul Daly:** Resources, Writing – review & editing. **Christopher E. Elgar:** Investigation, Writing – review & editing. **Daniel M. Mulvihill:** Resources, Writing – review & editing. **Andrew P. Abbott:** Conceptualization, Writing – review & editing, Funding acquisition. **Andrew Feeney:** Writing – review & editing, Supervision, Funding acquisition. **Paul Prentice:** Conceptualization, Methodology, Writing – original draft, Writing – review & editing, Supervision, Funding acquisition.

## Declaration of competing interest

The authors declare that they have no known competing financial interests or personal relationships that could have appeared to influence the work reported in this paper.
