# Supplementary material for: A mechanistic study identifying improved technology critical metal delamination from printed circuit boards at lower power sonications in a deep eutectic solvent
Source: Ultrason Sonochem. 2023 Nov 23;101:106701. doi: 10.1016/j.ultsonch.2023.106701 (PMC10711237; doi:10.1016/j.ultsonch.2023.106701)
Supplement: Supplementary data 1 [file mmc1.docx]

***Supplemental Material for:***

**A mechanistic study identifying improved technology critical metal delamination from printed circuit boards at lower power sonications in a deep eutectic solvent.**

Ben Jacobson^a^, Shida Li^a^, Rodolfo Marin Rivera^b^, Paul Daly^a^, Christopher E. Elgar^b^, Daniel Mulvihill^a^, Andrew P. Abbott^b^, Andrew Feeney^a^, Paul Prentice^a^.

*^a^James Watt School of Engineering, University of Glasgow, G12 8QQ*

*^b^School of Chemistry, University of Leicester, Leicester, LE1 7RH*

In this *Supplemental Material* document, sample data from the characterisation of sonotrode-generated cavitation in ethaline deep eutectic solvent (-DES), fig. 3 of the *Main Manuscript*, is presented and described. The figures below include representative shadowgraphic high-speed imaging (HSI) of cavitation development in the vicinity of the sonotrode-tip (from the Photron camera-Cavitar laser illumination perspective, fig. 2, *Main Manuscript*) and sample shockwave Passive Cavitation Detector (swPCD) data, recorded during 10 s sonciations, at three input powers; 60, 70 and 90%. These are key powers in terms of the structure displayed in the V_RMS_ versus input power plot, with 60% and 90% subsequently taken forward for the technology critical metal (TCM)-disk delamination observations. For the data presented here, no printed circuit board (PCB) section is present, as the primary purpose is a detailed characterisation of the cavitation in ethaline-DES, rather than TCM-delamination which is presented in the *Main Manuscript*. The Perspex disk on which the PCB section is mounted for delamination observations is present, however, 10 mm below the horn tip for consistency of positioning and immersion depth.

The general approach and data processing undertaken to arrive at a V_RMS_ versus input power plot, applicable to any ultrasonic source, in any liquid, follows the principles and procedures laid out in Yusuf *et al*, which characterised the cavitation generated by the same sonotrode used in the current study, in water [1].

**S.1 High-speed imaging and swPCD data of cavitation in Ethaline DES**


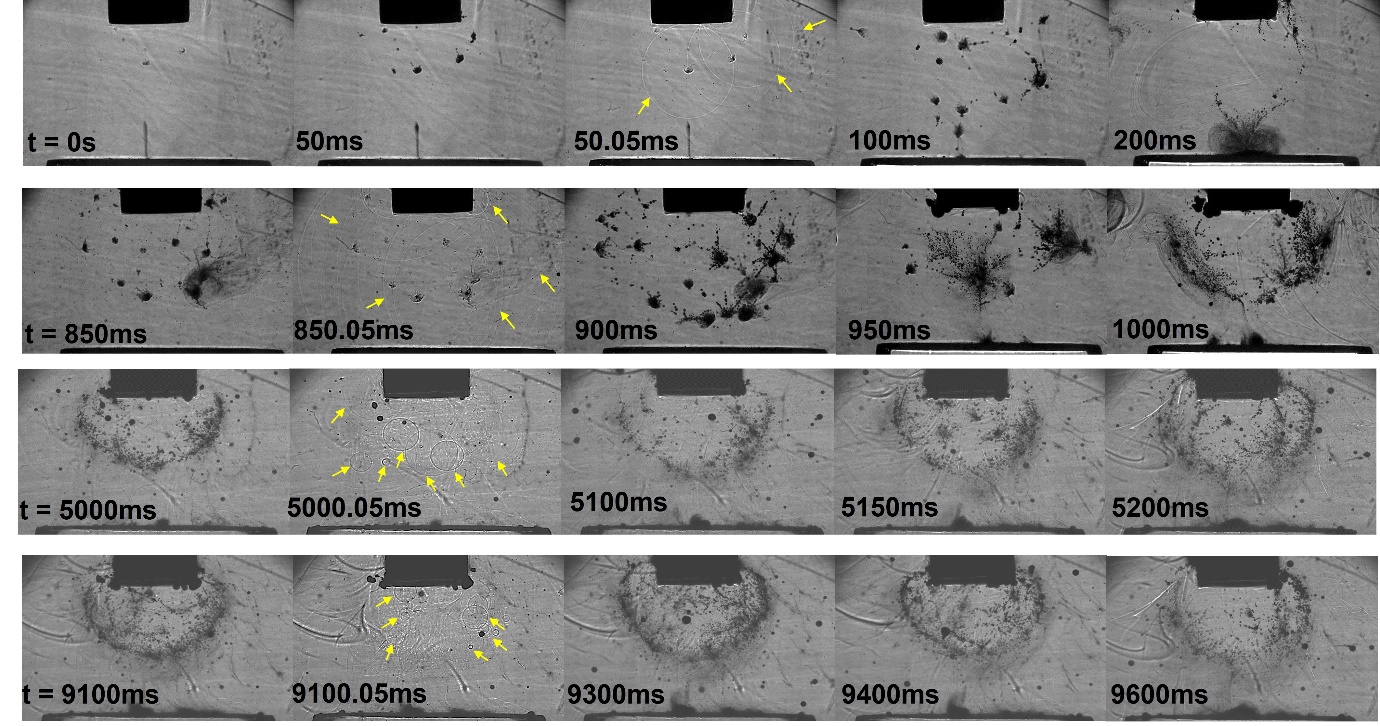
***S.1.1 At 60% input power***

*Fig. S.1: Representative high-speed imaging of the cavitation development in ethaline-DES at 60% input power over a 10s sonication. Bubble collapse shock waves captured by the shadowgraphic imaging capability are arrowed yellow, and scale is provided by the 6.4 mm diameter sonotrode tip.*

Fig. S.1 presents frames from a shadowgraphic HSI sequence recorded at 80000 frames per second (fps), broadly representative of cavitation structure evolution in the vicinity of the sonotrode tip, operating at 60% input power. The cavitation development is highly complex, particularly over the first 1000 ms of the sonication. Cavitation structures reminiscent of those previously reported in cleaning bath configurations [2] [3] and other ultrasound reactors [4] (central densely packed spherical bubble clouds, with bubble filaments extending radially outwards) are observed toward the end of the first second of the sonication. At 1000 ms, the cavitation has arranged itself into the bulbous structure characteristic of sonotrode sonications in viscous liquids [5-7]. Notably, this structure is maintained throughout the rest of the 10 s sonication, at this input power.


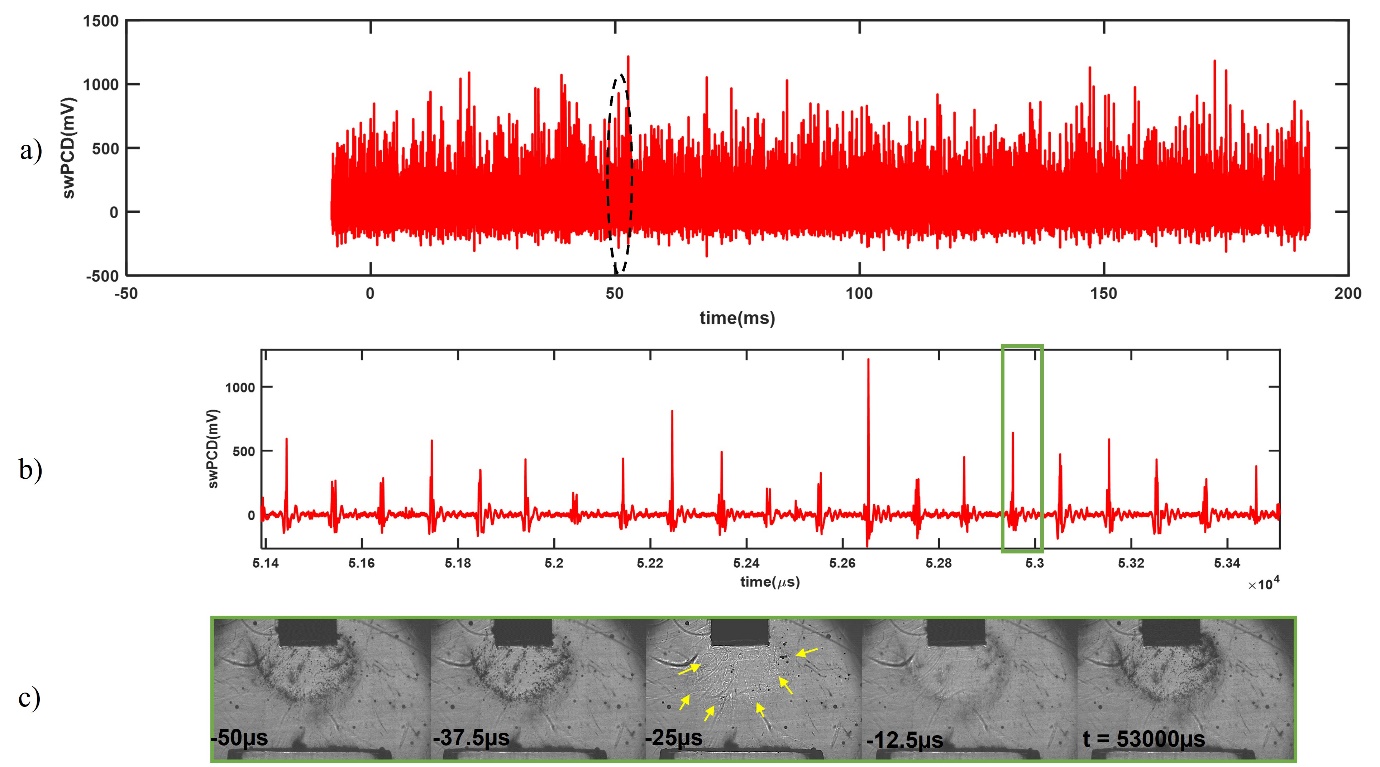
*Fig. S.2: (a) A 200 ms section of filtered swPCD data, recorded approximately 5 s into the 60% sonication in ethaline-DES, and (b) 2 ms of the data identified by the dashed-oval, on a shorter timescale, to reveal the bubble collapse shock wave characteristics. (c) Sample HSI extracted from the image-sequence, corresponding to the green box of (b).*

Fig S.2 (a) and (b) show swPCD data of the cavitation acoustic emissions, recorded approximately 5 s into the sonication captured by fig. S.1. As mentioned in the *Main Mansucript*, a filtering protocol was applied to reduce noise (low-pass < 10 MHz) and *f_0_* (high-pass > 20 kHz) and reveals bubble collapse shockwave content in the voltage-time domain. Fig S.2 (c) is HSI of the cavitation at the moment the shock wave emission highlighted by the green box, in fig. S.2 (b), was generated.

The important characteristics of the shock waves generated during a 60% input power sonication in ethaline-DES, are the shock wave timings at 2*T_0_*, where *T_0_* is the oscillation period of the sonotrode tip (= 50 µs, at the 20 kHz frequency of operation). This indicates that the cavitation activity of fig S.1 is exhibiting period-doubled behaviour, whereby bubble structures collapse for every other cycle of driving, at this input power. As demonstrated by Song *et al and* Yusuf *et al.*, this manifests in the spectrum of the cavitation noise as spectral peaks at *nf_0_/2*, where *n* is all integer values, fig. S.3 below. Namely, the *f_0_/2* subharmonic and its over-harmonics, as well as contributing to *f_0_* and its higher harmonics [1, 8].

The results shown in fig S.2 (a) and (b) also indicates variable shock wave amplitudes of between approximately 200 mV and 1000 mV are detected.


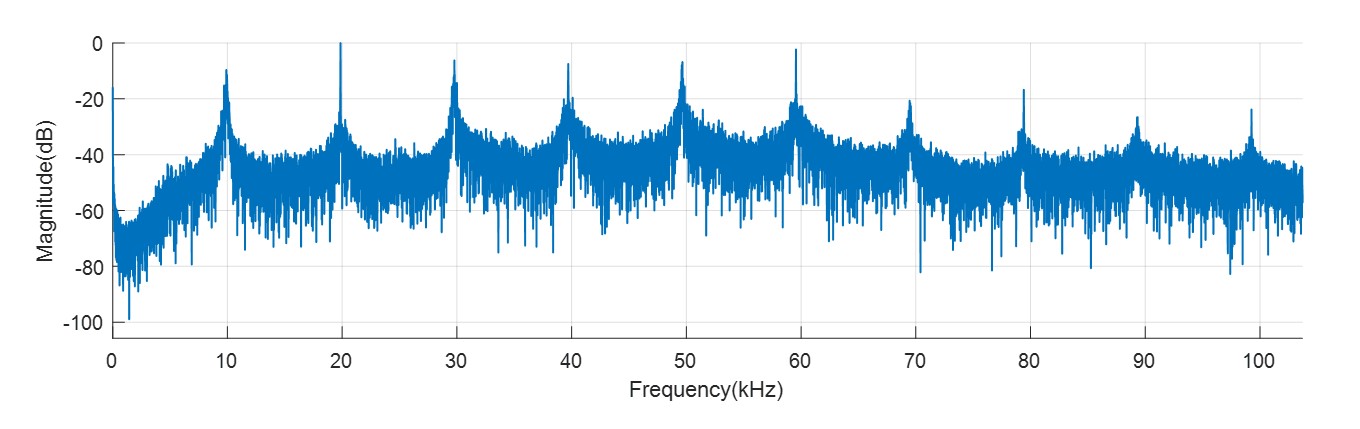
*Fig. S.3: Cavitation emission noise spectrum generated via application of a fast Fourier transform and Blackman window over the duration of the time signal, fig S.2 (a).*

***S.1.2 At 70% input power***

Figs S.4 – S.6 are equivalent to those described above, but at a sonotrode input power of 70%. The HSI of fig. S.4 indicates that the cavitation generated develops with some notable differences to that at 60%, as previously shown in fig. S.1.


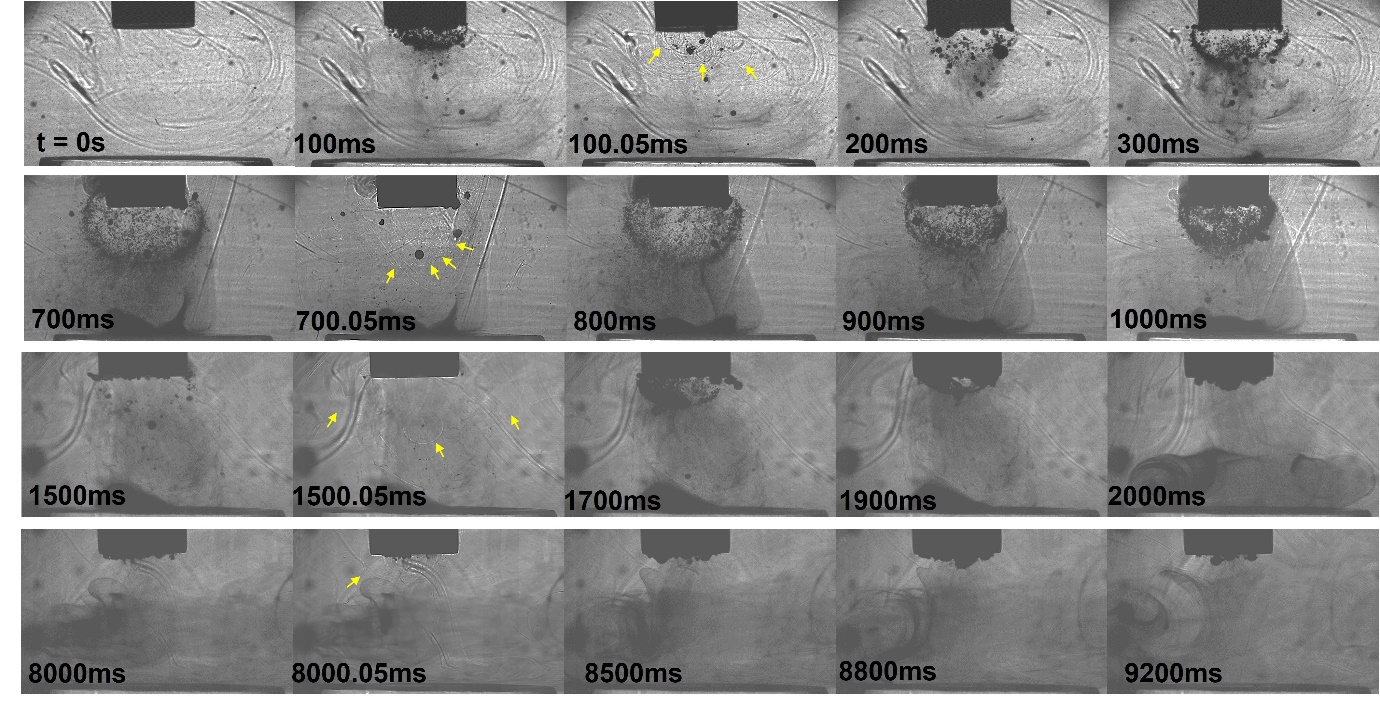
*Fig. S.4: Representative HSI of the cavitation development in ethaline-DES at 70% input power over a 10s sonication. Bubble collapse shock waves captured by the shadowgraphic imaging capability are arrowed yellow, and scale is provided by the 6.4 mm diameter sonotrode tip.*

The bulbous structure forms earlier in the sonication and, somewhat counter-intuitively, has a reduced radius-of-curvature around the tip, although with an apparent greater density of bubble clusters encapsulated. This structure is also accompanied by a fine mist of bubbles that ‘gradually’ pervades through the DES forming vortex structures on interacting with the surface of the Perspex disk under the tip, at 2000 ms, as shown in fig. S.4. The bubble mist does not appear to cavitate strongly, and no bubble-collapse shock waves are generated. It does, however, give an indication of the fluid flow via acoustic streaming within the ethaline-DES, at the slightly higher input power.

The main difference is that, unlike the sonication at 60%, the bulbous structure is not maintained throughout the sonication. From around 1500 ms, the main cavitation-activity recedes back into contact with the tip-surface, where it remains for the rest of the sonication. This phase of the cavitation development bears closer resemblance to sonotrode cavitation in less viscous liquids such as water [9, 10], although the cavitation oscillations are notably suppressed compared to a sonication at a comparable input power, in water. This could suggest that the liquid properties (including viscosity) are changing within a few seconds of sonications at higher input powers.

The swPCD data of fig. S.5 reveal the shock waves detected during a 200 ms duration, approximately 5 s into the 10 s sonication at 70% input power. Although there are a small percentage of shock waves with higher amplitudes than detected at 60%, fig. S.2, it can be seen that overall, the shock wave amplitude has actually reduced, at this higher input power.


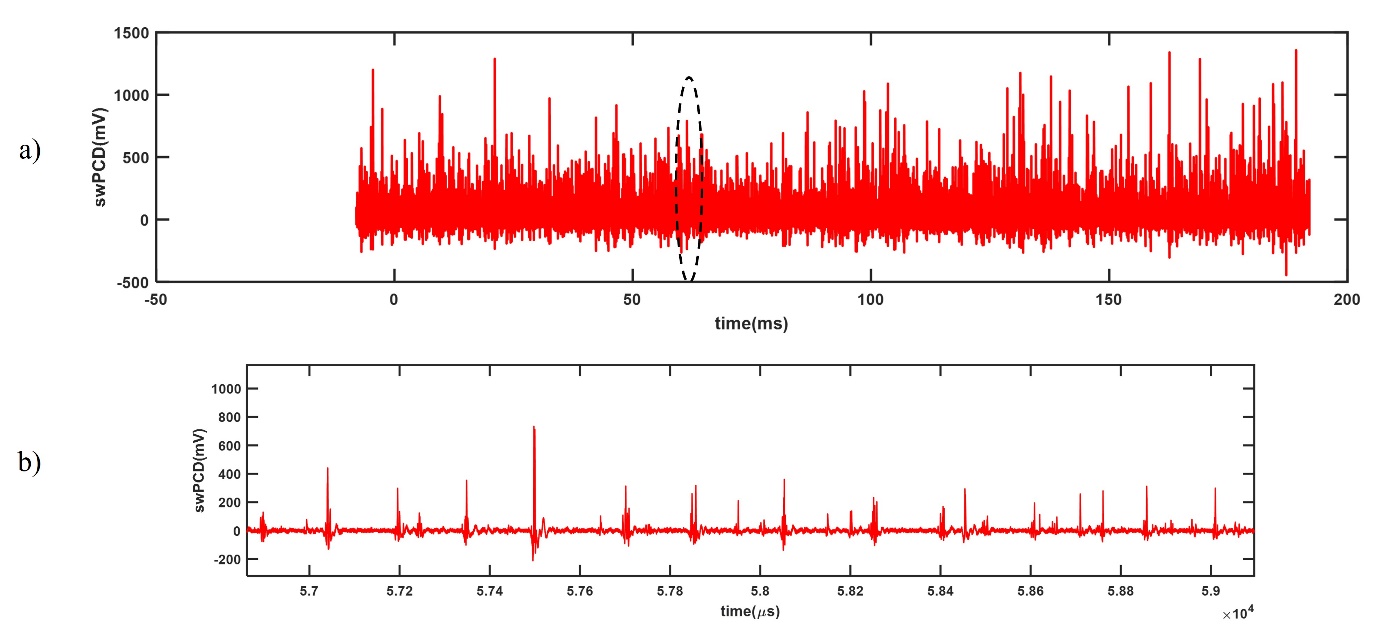
*Fig. S.5: (a) A 200 ms section of filtered swPCD data, recorded approximately 5 s into the 70% sonication in ethaline-DES, and (b) 2 ms of the data identified by the dashed-oval, on a shorter timescale, to reveal the bubble collapse shock wave characteristics.*

Moreover, the timings of the shock wave emissions at 70% have changed. Whereas at 60%, shock waves were detected at 2*T_0_*, irregular emissions at T_0_ and 3T_0_ are also apparent, at 70%, fig S.5 (b). This suggests that the cavitation in the DES during a sonication at 70% is transitioning toward period-tripled behaviour, as a higher-order subharmonic response.


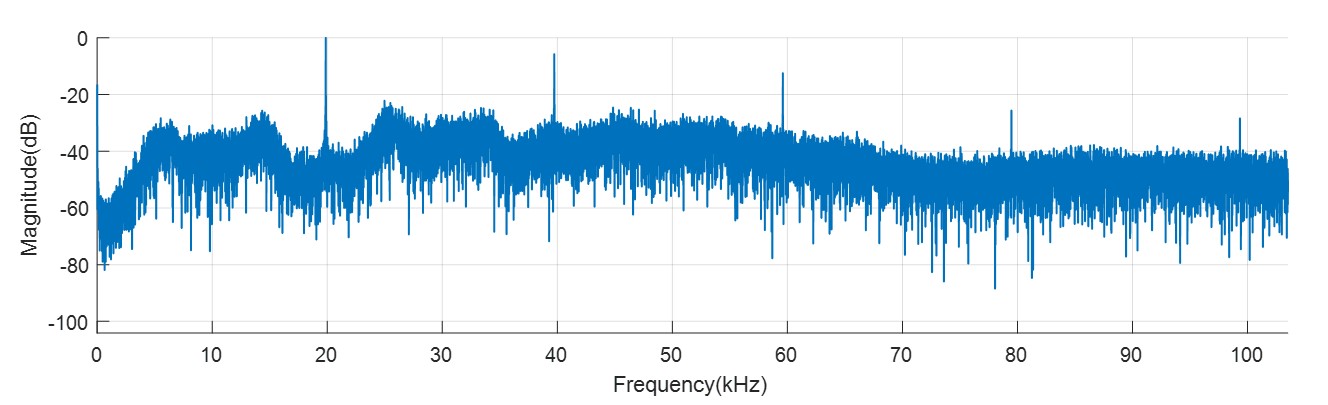
*Fig. S.6: Cavitation emission noise spectrum for sampled 200 ms signal at 70% input power in ethaline-DES.*

Indeed, the cavitation noise spectrum of this data reveals, low magnitude, broadened peaks at *nf_0_*/3, for all *n*, although > 50 kHz peaks between *nf_0_* values, are not apparent above the spectral floor (fig. S.6).

***S.1.3 At 90% input power***

Data for cavitation characterisation in DES at the second selected input power, taken forward for the TCM-delamination observations of the *Main Manuscript,* 90%, are presented below. The HSI of fig. S.7 demonstrates an enhancement in the development trends seen in the imaging data at 70%. The bulbous structure develops within the first 100 ms of the sonication, extending down to the surface of the Perspex disk at 200 ms. The receding of the main cavitation activity, back to the horn-tip is also accelerated, to within the first second of sonication. A fine bubble mist again reveals a vortex flow at the surface, from 1200 ms. A cavitating bubble cluster is also apparent, intermittently, in contact with the Perspex surface during the first few seconds, however, as with 70%, no bubble-collapse shockwaves were observed emitting from this. After that, the main cavitation is directly under the oscillating tip, and as would be expected, is more prominent than at the lower power of 70%.


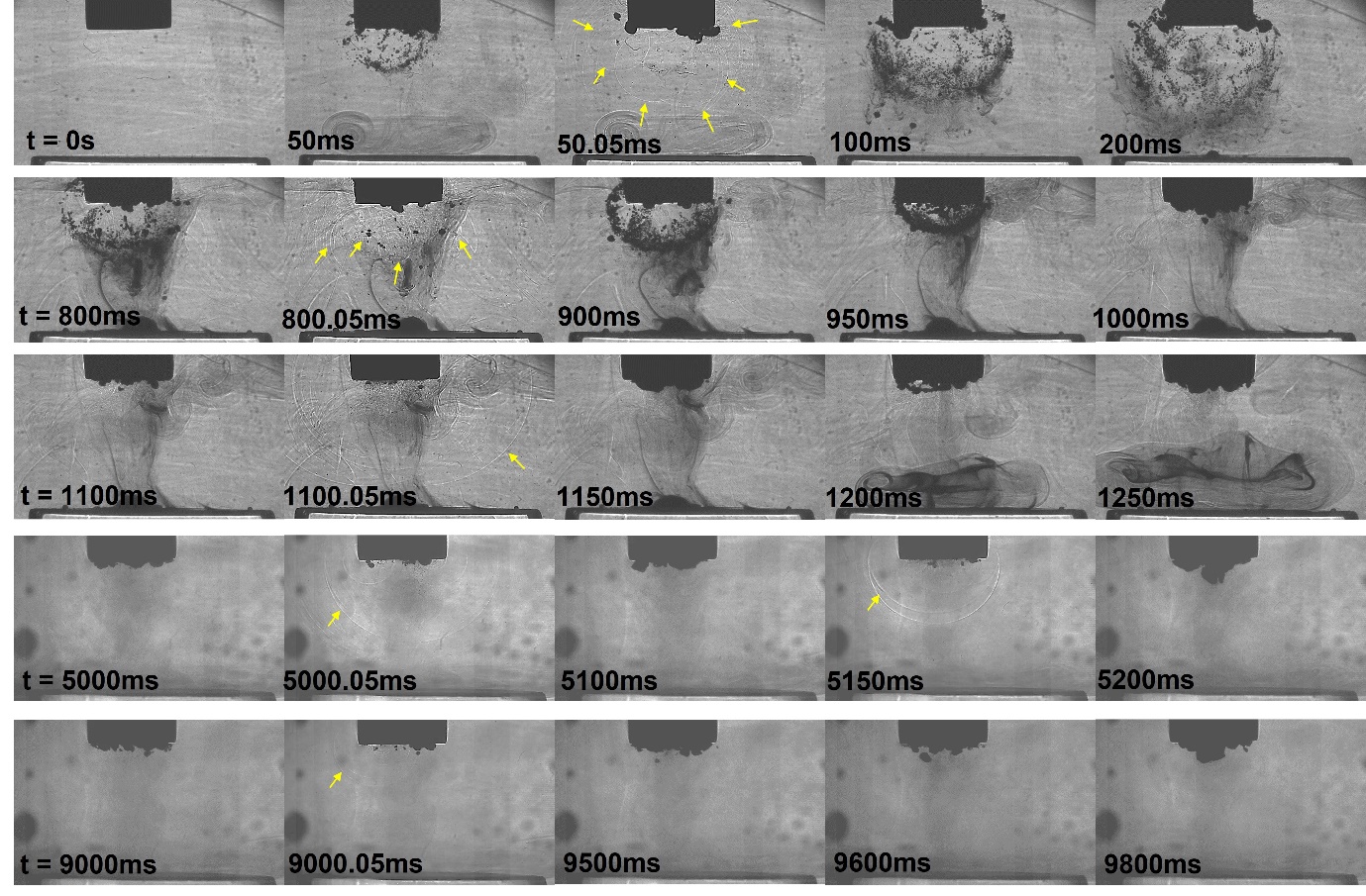
*Fig. S.7: Representative HSI of the cavitation development in ethaline-DES at 90% input power over a 10s sonication. Bubble collapse shock waves captured by the shadowgraphic imaging capability are arrowed yellow, and scale is provided by the 6.4 mm diameter sonotrode tip.*

The swPCD data at 90%, figs S.8 and S.9, reveal that a small number of high-amplitude shock waves were generated during the record duration. On the shorter timescale of fig. S.8 (b), smaller amplitude (< 100 mV) features occur at *T_0_*, suggesting the sub-clusters within primary cluster under the tip at this time of the sonication, are collapsing out of phase with others. Most of the higher amplitude shock waves, however, are detected a 4*T_0_* timings associated with period-quadrupled oscillation response. Accordingly, the spectrum of the cavitation emissions exhibits bumps at *nf_0_*/4, for all *n* until sensitivity falls below the noise floor (from ~70 kHz).


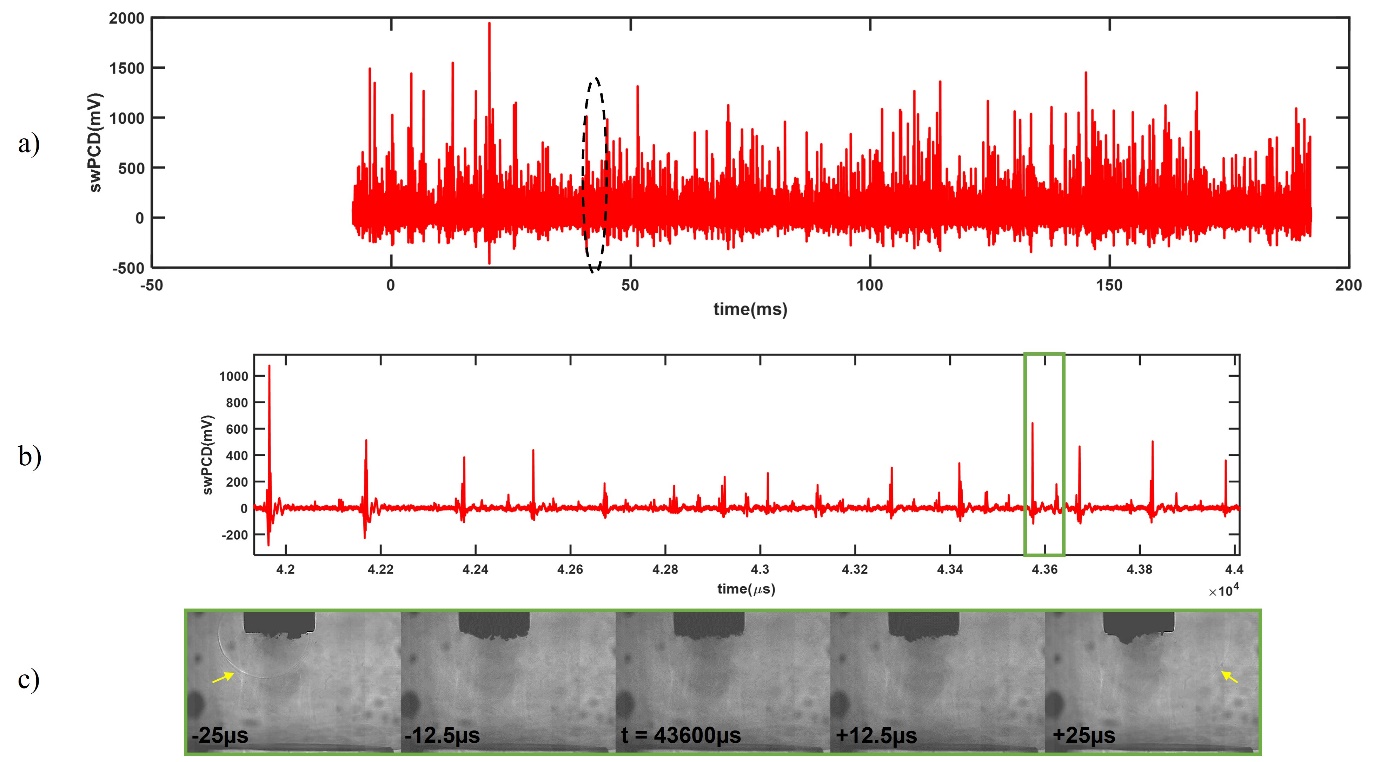
*Fig. S.8: (a) A 200 ms section of filtered swPCD data, recorded approximately 5 s into the 90% sonication in ethaline-DES, and (b) 2 ms of the data identified by the dashed-oval, on a shorter timescale, to reveal the bubble collapse shock wave characteristics. (c) Sample HSI extracted from the image-sequence, corresponding to the green box of (b).*

*Fig. S.9: Cavitation emission noise spectrum for sampled 200 ms signal at 90% input power in ethaline-DES.*

**S.2 V_RMS_ versus input power plots for Ethaline-DES and water**

The V_RMS_ versus input power plot for ethaline-DES, fig. 3 of the *Main Manuscript*, is reproduced as fig S.10, below, alongside an equivalent plot for water, fig S.11, generated within the experimental configuration used for this work, for compare and contrast purposes. Each data point represents five 200 ms sections of swPCD data collected approximately 5 s into a 10 s sonication at each power, as described above, with the error bars representing the standard deviation.

The main difference in experimental configuration between the water data of fig. S.11 and that presented in Yusuf *et al.* 2021 [1] is that for the latter, the swPCD was located directly below the tip, rather than to the side, as depicted in fig. 2 of the *Main Manuscript*. Nonetheless, the overall structure of the plot, including the V_RMS_ dips at key input power values of around 27% and 47% are very similar, indicating that the direction of acoustic detection does not influence the structure of these characterisation plots.

As described in Yusuf *et al*, and apparent in the DES cavitation data presented above, the dips in V_RMS_ with increasing power are due to the cavitation response transitioning from one subharmonic order (i.e. 2*T_0_* shock waves generating *nf_0_*/2 emissions, §S.1.1) to the next integer higher order (3*T_0_* shock waves generating *nf_0_*/3 emissions) [1]. Cavitation at transitioning input powers generate somewhat suppressed and irregular shock wave emissions, that reflect in V_RMS_ at that power. For cavitation driven at non-transitioning powers, comparatively consistent and regular shock wave emission lead to higher V_RMS_ values.

*Fig. S.10: Mean V_RMS_ from five 200 ms sonications at each input power, of the swPCD signal collected during sonications in ethaline-DES.*

*Fig. S.11: Mean V_RMS_ from five 200 ms sonications at each input power, of the swPCD signal collected during sonications in water.*

**S.3 Supplemental Material Discussion**

The comparison between ethaline-DES and water within the experimental configuration used for this work, suggests that each liquid has a unique characteristic V_RMS_ plot associated with it, for any given ultrasonic source. For sonochemical applications generally, the optimal power at which to drive the ultrasonic sources can thus be identified via hydrophone/cavitation detector measurements, sampling input power at sufficiently small increments. Although the HSI data presented here and in the *Main Manuscript* is useful in terms observing cavitation structure development and ultimately, identifying the underpinning mechanisms of TCM-delamination, it is not strictly necessary for guiding selection of optimal powers, either in this experimental configuration, or more generally.

In terms of the two input powers taken forward to the TCM-delamination work of the *Main Manuscript*, 60% was chosen as optimal, and 90% as a significantly higher, but non-optimal power. The delamination performance at each of these powers demonstrates the utility of V_RMS_ versus power plot, in real application terms, for the first time.

**Supplemental Material References:**

[1] L. Yusuf, M. D. Symes, and P. Prentice, "Characterising the cavitation activity generated by an ultrasonic horn at varying tip-vibration amplitudes," *Ultrason Sonochem,* vol. 70, p. 105273, Jan 2021, doi: 10.1016/j.ultsonch.2020.105273.

[2] F. Reuter, S. Lauterborn, R. Mettin, and W. Lauterborn, "Membrane cleaning with ultrasonically driven bubbles," *Ultrason Sonochem,* vol. 37, pp. 542-560, Jul 2017, doi: 10.1016/j.ultsonch.2016.12.012.

[3] T. Yamashita and K. Ando, "Low-intensity ultrasound induced cavitation and streaming in oxygen-supersaturated water: Role of cavitation bubbles as physical cleaning agents," *Ultrason Sonochem,* vol. 52, pp. 268-279, Apr 2019, doi: 10.1016/j.ultsonch.2018.11.025.

[4] C. J. B. Vian, P. R. Birkin, and T. G. Leighton, "Cluster Collapse in a Cylindrical Cell: Correlating Multibubble Sonoluminescence, Acoustic Pressure, and Erosion," *J. Phys. Chem.,* vol. 114, pp. 16416–16425, 2010.

[5] N. C. Eddingsaas and K. S. Suslick, "Evidence for a Plasma Core during Multibubble Sonoluminescence in Sulfuric Acid," *J. AM. CHEM. SOC.,* vol. 129, pp. 3838-3839, 2007.

[6] A. Thiemann, F. Holsteyns, C. Cairos, and R. Mettin, "Sonoluminescence and dynamics of cavitation bubble populations in sulfuric acid," *Ultrason Sonochem,* vol. 34, pp. 663-676, Jan 2017, doi: 10.1016/j.ultsonch.2016.06.013.

[7] I. Tzanakis, G. S. Lebon, D. G. Eskin, and K. A. Pericleous, "Characterizing the cavitation development and acoustic spectrum in various liquids," *Ultrason Sonochem,* vol. 34, pp. 651-662, Jan 2017, doi: 10.1016/j.ultsonch.2016.06.034.

[8] J. H. Song, K. Johansen, and P. Prentice, "An analysis of the acoustic cavitation noise spectrum: The role of periodic shock waves," *J Acoust Soc Am,* vol. 140, no. 4, p. 2494, Oct 2016, doi: 10.1121/1.4964633.

[9] A. Žnidarčič, R. Mettin, C. Cairós, and M. Dular, "Attached cavitation at a small diameter ultrasonic horn tip," *Physics of Fluids,* vol. 26, no. 2, 2014, doi: 10.1063/1.4866270.

[10] M. Khavari *et al.*, "Cavitation-induced shock wave behaviour in different liquids," *Ultrason Sonochem,* vol. 94, p. 106328, Mar 2023, doi: 10.1016/j.ultsonch.2023.106328.
